# Iritis

**Published:** 1904-02

**Authors:** 


IRITIS.

In conjunction with local treatment:

℞ Kalo iodid .................................... 3 drms.
Tongaline ...................................... 8 ozs.

M. et ft. sol. Sig.—A teaspoonful four times daily.

